# Related consistent lures increase the judgment of multiplication facts: Evidence using event-related potential technique

**DOI:** 10.3389/fnins.2023.1084309

**Published:** 2023-03-31

**Authors:** Yun Pan, Ji Shen, Lijuan Chen, Liangzhi Jia, Weiyu Tu, Huanyu Yang

**Affiliations:** School of Psychology, Guizhou Normal University, Guiyang, China

**Keywords:** digital cognition, multiplication, neighborhood consistency, N400, late positive component, event-related potential technique, related consistent lures

## Abstract

Simple multiplication errors are primarily shown in whether the lures are related to the operands (relatedness, such as 3 × 4 = 15 vs. 17) or whether the same decades are shared with the correct answers (consistency, such as 3 × 4 = 16 vs. 21). This study used a delayed verification paradigm and event-related potential technique to investigate the effects of relatedness and consistency in simple multiplication mental arithmetic for 30 college students in an experiment of presenting probes in auditory channels. We found that, compared to the related inconsistent lures, the related consistent lures showed significantly faster reaction time and induced significantly large amplitudes of N400 and late positive component. The findings suggest that related consistent lures are less affected by the activation diffusion of the arithmetic problem, and the credibility of being perceived as the correct answer is less; the lures related to operands and sharing the same decades with the accurate results can promote the judgment of multiplication mental arithmetic, and the results support the Interacting Neighbors Model.

## 1. Introduction

An analogy between arithmetic processing and sentence reading states that arithmetic comprises numbers and symbols to connect correct or incorrect, and sentences are made of words to convey potentially coherent information, suggesting similar potential cognitive processes (Salillas and Wicha, 2012; Dickson and Federmeier, 2017; Heidekum et al., 2019). Arithmetic problems are typically believed to be stored in interrelated memory networks (Campbell, 1997; Domahs and Delazer, 2005; Verguts and Fias, 2005a). This is similar to the existing cognitive neural mechanism of lexical language processing: lexical-semantic connections affect the cognitive processing of sentences (Rhodes and Donaldson, 2008; Swaab et al., 2012). Hence, a semantic relationship may exist between operands and probes. Considering this perspective, neighborhood consistency is introduced to investigate whether the multiplication arithmetic solution is affected by the difference in the intensity of semantic connections between arithmetic operands and probes.

Domahs et al. (2007) stated that adults are affected by the relatedness and consistency of presenting probes when dealing with multiplication facts. Relatedness refers to whether the probes are related to an operand of the arithmetic problem, and whether the correlation is related. For example, the lure 18 is related to the operand of arithmetic 4 × 6 through 6 (3 × 6 = 18), and vice versa. Consistency refers to whether the presented lures have the same decade digits as the correct result and is consistent with having the same decade. For example, the lure 12 has the same decade digit “1” as the correct result 18 for problem 3 × 6; otherwise, it is inconsistent. Additionally, Domahs et al.’s (2007) study showed that when the probes (lures) are related to the operand and share the same decade digits as the correct result, they are related to consistent trial times; when the wrong lures are related to the operand but do not share the same decade digits as the correct result, they are related to inconsistent trial times. Several studies have shown that the response time of related consistent lures is faster and more accurate than that of related inconsistent ones (Verguts and Fias, 2005a;^57,11^; Rotem and Henik, 2015). Therefore, the difference in reaction time and accuracy between related consistent and related inconsistent lures is defined as neighborhood consistency, used to measure the intensity of semantic connection between arithmetic operands and probes.

Thus far, the discussion on neighborhood consistency primarily focuses on the Network Interference Model (NIM) and the Interacting Neighbors Model (INM) (Campbell, 1987; Campbell, 1995; Verguts and Fias, 2005a). Both theories believe that arithmetic is stored in an interrelated network, and the representation of an arithmetic problem (e.g., 3 × 6) both activates the correct answer (18) and also automatically activates the related lures [e.g., 12 = (2 × 6), 24 = (4 × 6)]. However, both have different views on how arithmetic problems and related lures affect multiplication mental arithmetic judgment. Campbell (1987) proposed the NIM, emphasizing the interaction between coding and extraction. Activation diffusion from operands activates the answer and the lures related to each operand, and the retrieval process distinguishes between candidate sets to find the most active value, limiting the speed and accuracy of retrieval. Additionally, the activation of different probes increases the competition to retrieve the probes, leading to the wrong lures with the same decade digits as the answer, a longer reaction time, and a higher error rate. Verguts and Fias (2005a) proposed the INM, suggesting that multiplication problems and probes are semantically related. Arithmetic retrieval involves two-layer processing that promotes cooperation and suppresses competition. If the lures are related to the operands and have the same decades, it promotes the response to the probes, while if they have different decades, it suppresses the response to the probes.

Since neighborhood consistency was proposed, researchers have attempted to verify it from different viewpoints. Verguts and Fias (2005b), using natural numbers from two to nine as materials, used arithmetic naming tasks to investigate the effect of related lures on mental arithmetic. The results showed that participants’ reaction time increased when related arithmetic problems were practiced compared with the unpracticed group. Subsequently, the study analyzed the error rates of 36 arithmetic multiplication among 44 adults and showed that operand-related errors were more likely to involve decade consistent lures than decade inconsistent, and the proportion of these errors increased with age (Domahs et al., 2006). The researchers adopted the production paradigm (participants manually entered arithmetic results), selected adults as participants, included numbers 2–9 as operands for materials, and asked them to memorize 16 equivalent (such as 2 # 2 = 65) and non-equivalent formulas (such as 3 # 2 = 75) to exclude the numerical relationship between operands and probes, and directly examined the effect of neighborhood consistency on arithmetic memory. The results showed that compared with the accuracy of related inconsistent lures (42%), the accuracy of related consistent (72%) is higher, indicating that related consistent lures can promote the learning and development of arithmetic memory (Campbell et al., 2011). Rotem and Henik (2015) selected children with and without mathematical learning disabilities (MLD) as participants, using numbers from two to nine as materials and an arithmetic verification paradigm to investigate the sensitivity of different participants to multiplicative arithmetic-related and consistent numerical features. They found that children were first sensitive to operand-related numerical features (children without MLD started in Grade 3; children with MLD in Grade 8) and also to decade-consistent numerical features (normal children started in Grade 4 and began to mature in Grade 6). Domahs and his colleagues used event-related potential (ERP) technology to study neighborhood consistency in mental arithmetic. They selected numbers from two to nine as operands and adopted an arithmetic verification paradigm to investigate their effects on arithmetic judgment by directly manipulating the relatedness and consistency of simple arithmetic. The results showed that related inconsistent lures induced larger N400 components and late positive components (LPC) than related consistent ones (Domahs et al., 2007). N400 is a negative waveform appearing in the central and parietal regions, usually caused by the violation of semantic expectation and the relevance between words (Rhodes and Donaldson, 2008). LPC is a positive slow wave component related to semantic processing, stimulus recognition, and credibility identification (Polich, 2012), the greater the amplitude of LPC, the lower the subjective credibility of individuals to judge the stimulus as the answer. In summary, people are easily affected by relatedness and consistency when dealing with multiplication facts.

The above studies had certain limitations in their design and methods. Verguts and Fias (2005b) attempted to manipulate consistency by including or excluding related problems, which led to the manipulation of extraction to induce forgetting (Phenix and Campbell, 2004; Campbell and Thompson, 2012). Additionally, although Domahs et al. (2007) and Rotem and Henik (2015) directly manipulated relatedness and consistency, they did not deny the influence of distance effect on the probes. Ashcraft and Stazyk (1981) found that the reaction time to the wrong lures of multiplication races with increased numerical distance from the answer. The “split effect” in mental multiplication refers to the phenomenon where individuals have faster reaction times and higher accuracy rates when the numerical split between the presented answer and the correct answer is greater, and slower reaction times and lower accuracy rates when the numerical split is smaller (Isabel Núñez-Peña and Escera, 2007). For example, the arithmetic 3 × 6 = 22 is easier to judge and has a higher accuracy rate than 3 × 6 = 20. Related studies suggest that the effect of numerical split on mental arithmetic judgments is mainly due to individuals using two different retrieval strategies to compare the presented answer and the correct answer to solve arithmetic problems. When the proposed answer is close to the correct answer, the subject may use a more detailed whole-calculation strategy to achieve accurate calculation. However, when the proposed solution is far from the correct solution, the subject may not need to obtain accurate calculation and can use a more easily and quickly estimated plausibility-checking strategy to judge the truth of the answer (Duverne and Lemaire, 2005; El Yagoubi et al., 2005). A study showed that relatedness and distance effects have an additive effect on arithmetic reaction time; that is, the closer the wrong lures are to the answer, the more time it takes to reject the judgment, and more time is needed if the probes are related to one of the two operands (Ashcraft, 1992). In other words, the closer the wrong lures are numerically to the answer, the more time it takes to reject the judgment, and more time is needed if the lures are related to one of the operands. Regarding methods, most of the above studies use reaction time as an analysis index; however, reaction time and electrophysiological indexes may reflect different analysis processes for individuals. Studies have shown that reaction time reflects the late process of decision competition (Duncan-Johnson and Kopell, 1981), while the electrophysiological index occurs earlier than explicit reaction, and its latency is usually regarded as the end point of individual analysis (McCarthy and Donchin, 1981), which is not affected by the reaction preparation process. Therefore, the behavioral response occurs later than the electrophysiological response in most studies, and the lag of this response time has been found in some studies (Brown and Hagoort, 1993; Holcomb, 1993; Niedeggen and Rösler, 1999). Research on neighborhood consistency has primarily focused on behavioral experiments; hence, the study of electrophysiology must be further discussed.

Additionally, few studies on neighborhood consistency in multiplication present probes by auditory channel. Studies have shown that humans may have different cognitive processes when dealing with external information from different channels. For example, some studies have found that auditory learning visual test conditions produce more false memory than visual learning test conditions in learning-recognition paradigm tasks (Gallo et al., 2001; Pierce et al., 2005). In Zhu et al.’s (2019) study, functional magnetic resonance imaging (fMRI) was used to investigate the effects of information learning and testing in different channels on memory intensity by manipulating the sensory patterns of vocabulary presentation (auditory and visual) of college students. The results showed that compared with the visual learning visual test group, the auditory learning visual test group showed less representation matching between coding and extraction in the visual cortex and greater semantic activation in the temporal pole, indicating the similarity between coding and extraction promoted memory retrieval. To determine the influence of arithmetic presentation channel on human mental arithmetic and its cognitive process, Dickson et al. (2018) used the numbers two to nine as the materials by delayed verification paradigm and ERP technology under the sensory mode (vision) of constant lures presentation to investigate whether the arithmetic problem presentation channel affects the brain’s response to multiplication problem-solving. The results showed that for visual presentation of arithmetic problems, the answers induced apparent P300 components and the lures induced LPC, while the auditory answers could not induce P300 components and the lures induced N400 components, indicating that phonetic numbers have stronger semantic activation and the brain has different processes arithmetic information at different representation. The effects of audio and visual channel differences on mental arithmetic multiplication are primarily explained by Transfer-appropriate Processing (Morris et al., 1977) and Encoding Specificity Principle (Tulving and Thomson, 1973). Both believe that memory is the ability to encode, store, and retrieve information; when there is a substantial similarity between coding and retrieval (i.e., the initial acquisition channel of information is the same as the test channel), it tends to induce correct memory. However, on the contrary, it is more likely to produce false memory, which is supported by relevant behavioral studies (Roediger and Guynn, 1996; Smith and Hunt, 1998). Studies show that the difference between the initial learning and test channels increases the memory retrieval difficulty, suggesting that learning and memorizing multiplication arithmetic mainly through verbal strategies (oral repetition) (Dehaene, 1992; Zhou and Dong, 2003; Zhou et al., 2007; Cheng et al., 2017), auditory channels present arithmetic probes, leading to the representation of arithmetic problems to produce greater activation diffusion in the memory network, and thus affecting the extraction of arithmetic probes.

Hence, to the best of our knowledge, this is the first study to utilize the ERP technique to explore the influence of relatedness and consistency of lures in an auditory channel on multiplication mental arithmetic. Under the premise that the distance between the answer and the lures is constant, we manipulate the relatedness and consistency of multiplication arithmetic lures to verify whether the activation and diffusion of multiplication algorithms stored in long-term memory in the memory network will affect the related lures in the experiment, and explore the impact of the same decades on arithmetic cognitive neural activity. This study recorded the reaction time and accuracy of different participants to multiplication facts in the experiment, and by observing different ERPs, it determined the influence of relatedness and consistency in multiplication mental arithmetic and whether it is sensitive to the present form of the probes. First of all, on the basis of previous studies, because there is a stronger semantic activation relationship between arithmetic questions and answers in the auditory channel, we predict that there is no difference in the activation and diffusion of event-related potentials between adjacent and non-adjacent answers. Secondly, we also predict that arithmetic extraction includes two-layer processing that promotes cooperation and suppresses competition, the related consistency effect appears in simple multiplication mental arithmetic under the presence of auditory channels, and the N400 and LPC amplitudes induced by related consistent lures are larger than those induced by related inconsistent ones.

## 2. Materials and methods

### 2.1. Participants

The sample size was estimated by G*Power 3.1 software (Faul et al., 2009), wherein *f* = 0.25 (medium effect; Muller, 1989), *α* = 0.05, Power = 0.80, and the smallest sample size was 24. Thirty college students were recruited for the experiment, all of whom were right-handed, had normal or corrected vision, had no cognitive nerve damage (including a history of brain injury), and were currently not using psychoactive drugs. As the electroencephalogram (EEG) artifacts of two participants were higher than 25%, they were excluded from the study (6.7%). There were 28 valid participants (13 males) with an average age of 21.39 years [Standard Deviation (*SD*) = 1.95]. They were required to sign an informed consent form before the experiment began and received a specific reward at the experiment end. The experiment was approved by the Ethics Committee of the College of Psychology of Guizhou Normal University.

### 2.2. Materials

The experimental materials are 18 multiplication arithmetic problems (Domahs et al., 2007; Rotem and Henik, 2015). To exclude the influence of the formula rule (N × 0 = 0, N × 1 = 1) on the experimental results (Campbell and Metcalfe, 2007), the formal experiment does not contain zero and one problems. The range of operands therefore includes natural numbers from two to nine. The stimuli characters in the experiment were all in Arial font, with a size of 1.24° × 1.74°. The probes for each multiplication fact comprised one correct answer and four wrong lures. Among them, in the wrong lures, the related lures only use the product distance of one from the operand of the arithmetic problem [e.g., 16 = 8 × (3–1)], and the distance between the unrelated lures and the correct answer is equal. Additionally, to avoid the “five rule” (one of the operands is five, the last digit of the result must be five or zero) in a multiplication set, if the wrong lures less than the correct result contain five or zero, the lures t greater than the correct result also includes five or zero (e.g., 7 × 5 = 30 or 40).

### 2.3. Design

The two-factor internal design of the experiment is 2 (relatedness: related lures, unrelated lures) × 2 (consistency: consistent lures, inconsistent lures). Among them, related consistent indicates that the lures are related to one of the operands of the arithmetic problem, and the decade is similar to the correct answer (e.g., 3 × 6 = 12), in contrast to related inconsistent (e.g., 3 × 6 = 24). Unrelated consistent indicates that the lures are not related to the operands of the arithmetic problem; however, the decade digits are the same (e.g., 3 × 6 × 14), and the opposite is true for unrelated inconsistent (e.g., 3 × 6 × 22). The dependent variables were reaction time, correct rate, N400, and LPC amplitude.

### 2.4. Procedure

Using Psychopy 3.0 for programing, the experimental materials were all displayed on 23.5inch computer monitor with a 1920 × 1,080 resolution and refresh rate of 60 Hz. Using the delayed verification paradigm, each trial begins with an eye point of 800 ms (ms) “*” (0.80° × 0.74°). Subsequently, two operands were presented in turn, each 450 ms, with a blank screen (ISI, Interstimulus Interval) of 250 ms between the operands, and the second operand appeared together with the multiplication symbol (e.g., × 9, size 2.45° × 1.74°). The probes then appeared in the auditory behind the blank screen of the 550 ms and continue to present the 350 ms for a certain period. The probes were followed by a delay of 650 ms as a blank screen period for ERP data collection, and then the prompt “?” appeared on the screen for at least 350 ms. Subsequently, the participants were asked to press the “?” symbol as soon as it appeared.

**Figure 1:**
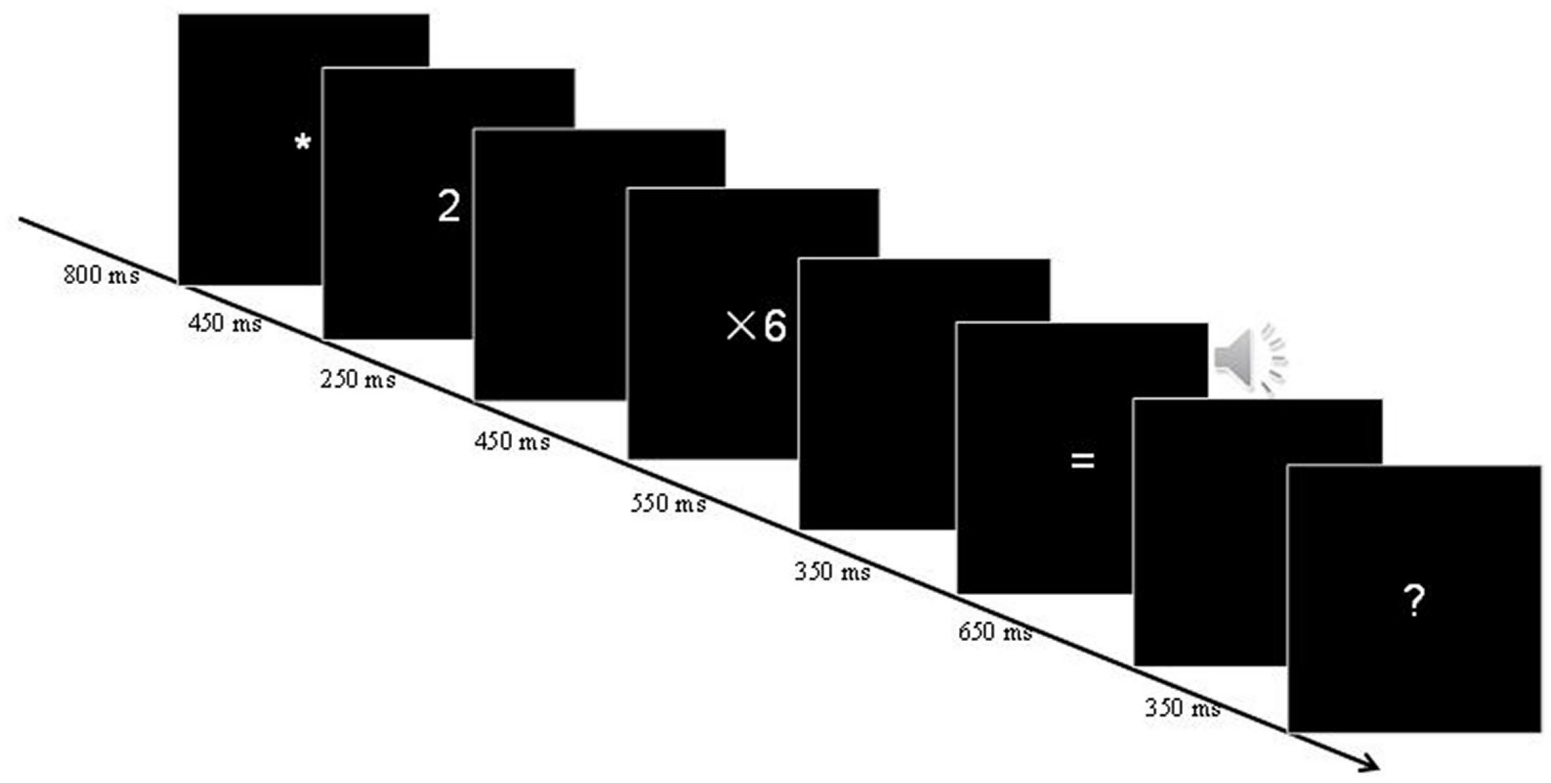
Illustration of the in this study. All operands are presented as visual stimuli, arithmetic lures are presented as auditory stimuli, and delayed verification tasks are used when “?” appears, the participants will be able to respond to the right or wrong of the arithmetic results.

If the probes were correct, half of the participants were asked to press “←” with the index finger of the right hand and press “→” with the middle finger of the right hand if wrong. For the remaining participants, the buttons with correct and wrong lures were opposite. All stimuli were presented in the center of the screen to avoid interference with EEG collection caused by eye movements. The delayed verification response prevents artifacts caused by keystrokes from interfering with EEG records, with a one-second trial interval remaining between each trial, during which the participants are allowed to blink. All stimuli were displayed on a computer monitor about 70 cm from the participants. The specific experimental flow is shown in Figure 1.

The experiment comprised two parts: the practice experiment and the formal experiment. There were six trials in the practice experiment, and the participants began the formal experiment only when they understood the experiment requirements. To avoid the influence of the practice effect in the experiment, some arithmetic problems of the practice were separate from the materials of the formal experiment (such as 3 × 8 = 16). The formal experiment randomly presented 18 arithmetic sets, each presented 24 times, wherein the correct answer is presented 12 times, and each condition of the wrong lures is presented three times, a total of 432 trials. The formal experiment comprised six blocks, each composed of 74 trials, with a short break of 1–3 min between each block, and the whole experiment was completed in approximately 50 min.

### 2.5. EEG recording and analysis

Using the Neuroscan4.5 analysis and recording system and Synamps2.0 amplifier, a 64-lead non-invasive electrode cap distributed following international standards 10–20 was selected to record EEG data. The Cz electrode on the top of the head is used as an online reference, and the neutral point of the Fz and FPz electrodes is grounded. To monitor blinking and horizontal eye movement, vertical eye movement was recorded through the electrodes above and below the left eye, and horizontal eye movement was recorded by two electrodes in the outer corner of both eyes. The sampling rate is 500 Hz, the bandpass filter is 0.1–30 Hz (Tanner et al., 2015), and all the electrode impedances are less than 5 kΩ.

The EEG data were analyzed using EEGLAB 9.0 and ERPLAB 14.0 for offline data processing. Neuroscan4.5 was used to record and collect EEG signals, and using a 64-channel amplifier with a sampling frequency of 500 Hz, EEG activity was recorded with reference to the left mastoid, and the average values of the left and right mastoids were re-referenced offline (Brain Products, Gilching, Germany). The recorded EEG data were filtered (0.01–30 Hz; slope 12 dB/oct; zero phase) and segmented by 100 ms before the start of stimulation. Using ICA (Independent Component Analysis) to remove artifacts such as blinks, eye movements, and muscles. All data are baseline corrected according to the average voltage within 100 milliseconds before the start of the stimulus, and then the waveforms under all conditions are averaged. Before the superposition average, the trials with amplitudes other than ±100 μV were excluded, only the correct trials were superimposed, and the average number of superimposed segments of all participants under each condition was more than 30. Considering this study’s aims and Domahs et al.’s study (2007), FZ, FC5, FC6, CZ, C3, C4, and PZ in the frontal lobe, parietal lobe, and central area of the brain were selected using 360–540 ms and 550–700 ms as time windows, and the peak amplitude of N400(360-540 ms) and LPC (550–700 ms) were measured, respectively. The *p* values of main effects and interactions that did not conform to the spherical hypothesis were corrected by the Greenhouse–Geisser method.

## 3. Results

### 3.1. Behaviors

When excluding the reaction errors under various conditions and reactions, except ±3 SD, the elimination rate was 4.84%. The correct rate of all participants was 0.98 ± 0.03 (M ± SD), and the correct average rate of each participant was higher than 92%; hence, the data is not excluded. Repeated measurement variance analysis of 2 (relatedness: related lures, unrelated lures) × 2 (consistency: consistent lures, inconsistent lures) was performed for reaction time and accuracy.

#### 3.1.1. Response time

The results showed that the primary effects of relatedness was not significant [*F*(1,27) = 0.18, *p =* 0.679], consistency was not significant [*F*(1,27) = 0.42, *p* = 0.52], and the interaction between them was significant [*F*(1,27) = 11.33, *p* = 0.002, η_p_^2^ = 0.002]. Further, simple effect analysis showed that under related conditions, the average reaction time of consistent lures (340.39 ms ± 150.45) was significantly faster than inconsistent ones [(361.35 ms ± 161.16, M ± SD, same below), *F*(1,27) = 7.55, *p* = 0.011, η_p_^2^ = 0.22]. Under the consistent conditions, the average reaction time of related lures (340.39 ms ± 150.45) was significantly faster than that of unrelated ones [360.22 ms ± 162.54; *F*(1,27) = 7.56; *p* = 0.011; η_p_^2^ = 0.22]. Under the inconsistent condition, there was no significant difference in the reaction time between the related lures and the unrelated ones; *F*(1,27) = 2.61; *p* = 0.12.

#### 3.1.2. Accuracy

The main effect of relatedness was not significant [*F*(1,27) = 2.90, *p* = 0.10]. The main effect of consistency was not significant [*F*(1,25) = 1.49, *p* = 0.23]. The interaction between relatedness and consistency was not significant [*F*(1,27) = 0.82, *p* = 0.78]. The reaction time and accuracy under various conditions are shown in Table 1.

**Table 1:** Reaction time and accuracy under four conditions (M ± SD).

Index	Rel-cons*	Rel-incons**	Unrel-cons^#^	Unrel-incons^##^
RT (ms)	340.39 ± 150.45	361.35 ± 161.16	360.22 ± 162.54	346.84 ± 149.47
Accuracy (%)	97.62 ± 3.63	98.01 ± 3.31	98.02 ± 2.98	98.55 ± 3.20

*Related consistent; **Related inconsistent; ^#^Unrelated consistent; ^##^Unrelated consistent.

### 3.2. Event-related potentials

#### 3.2.1. N400

**Figure 2:**
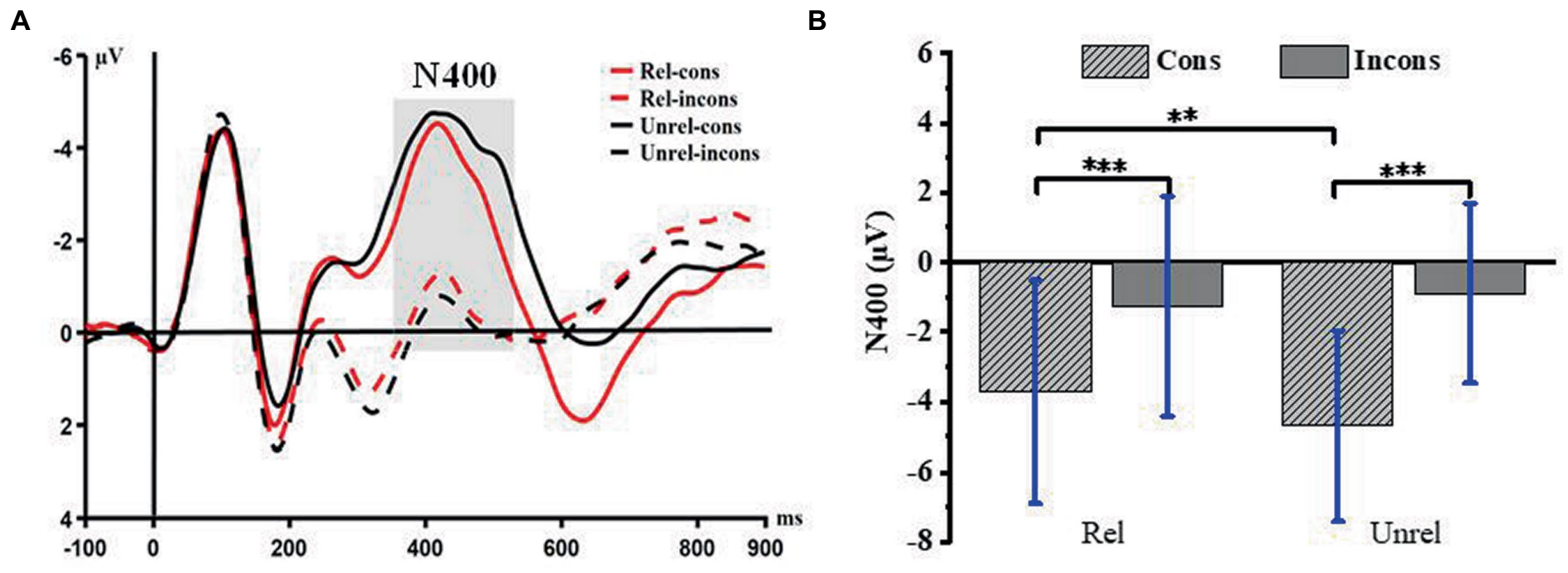
**(A)** The N400 component time-locked to the four conditions. Event-related potentials (ERPs) were calculated by averaging the data at the electrodes of Fz, FC5, FC6, CZ, C3, C4, and PZ. **(B)** Histogram in conditions, bars represent standard deviation of the mean. **p < 0.01, ***p < 0.001. Rel, related; Unrel, unrelated; Cons, consistent; Incons, inconsistent.

The repeated measurement variance analysis of 2 (relatedness: related lures, unrelated lures) × 2 (consistency: consistent lures, inconsistent lures) was performed on the average amplitude of N400 in the time window of 360–540 ms. The results showed that the main effect of relatedness is not significant [*F*(1,27) = 0.98, *p* = 0.33, η_p_^2^ = 0.04], and there was no significant difference in the average amplitude between the related lures (−2.50 μV ± 3.02, M ± SD, same below) and the unrelated ones (−2.79 μV ± 2.50). The main effect of consistency was significant [*F*(1,27) = 102.44, *p* < 0.001, η_p_^2^ = 0.79], and the average amplitude of N400 induced by consistent lures (−4.20 μV ± 2.83) was significantly higher than that induced by inconsistent ones (−1.08 μV ± 2.72). The interaction between relatedness and consistency was significant [*F*(1,27) =12.97, *p* = 0.001, *η_p_^2^* = 0.33]. Further simple effect analysis showed that under related conditions, the average amplitude of N400 induced by consistent lures (−3.71 μV ± 3.19) was significantly larger than that induced by inconsistent ones (−1.27 μV ± 3.15), *F*(1,27) = 43.17; *p* < 0.001; *η_p_^2^* = 0.62. Under unrelated conditions, the amplitude of N400 evoked by consistent lures (−4.69 μV ± 2.74) was significantly higher than that induced by inconsistent ones (−0.89 μV ± 2.58); *F*(1,27) = 116.79; *p* < 0.001; η_p_^2^ = 0.81. Under the consistent conditions, the average amplitude induced by unrelated lures (−4.70 μV ± 2.74) was significantly higher than that induced by the related ones (−3.71 μV ± 3.19); *F*(1,27) = 116.79; *p* < 0.001; η_p_^2^ = 0.81. This shows that the activation diffusion of arithmetic questions will activate not only the correct answers but also the related wrong lures, and the extraction of related wrong lures may require more cognitive resources. Under the inconsistent condition, there is no significant difference between related lures and unrelated [*F*(1,27) = 1.14, *p* = 0.30]. Detailed waveforms and bar charts are shown in Figure 2.

#### 3.2.2. Late positive components

**Figure 3:**
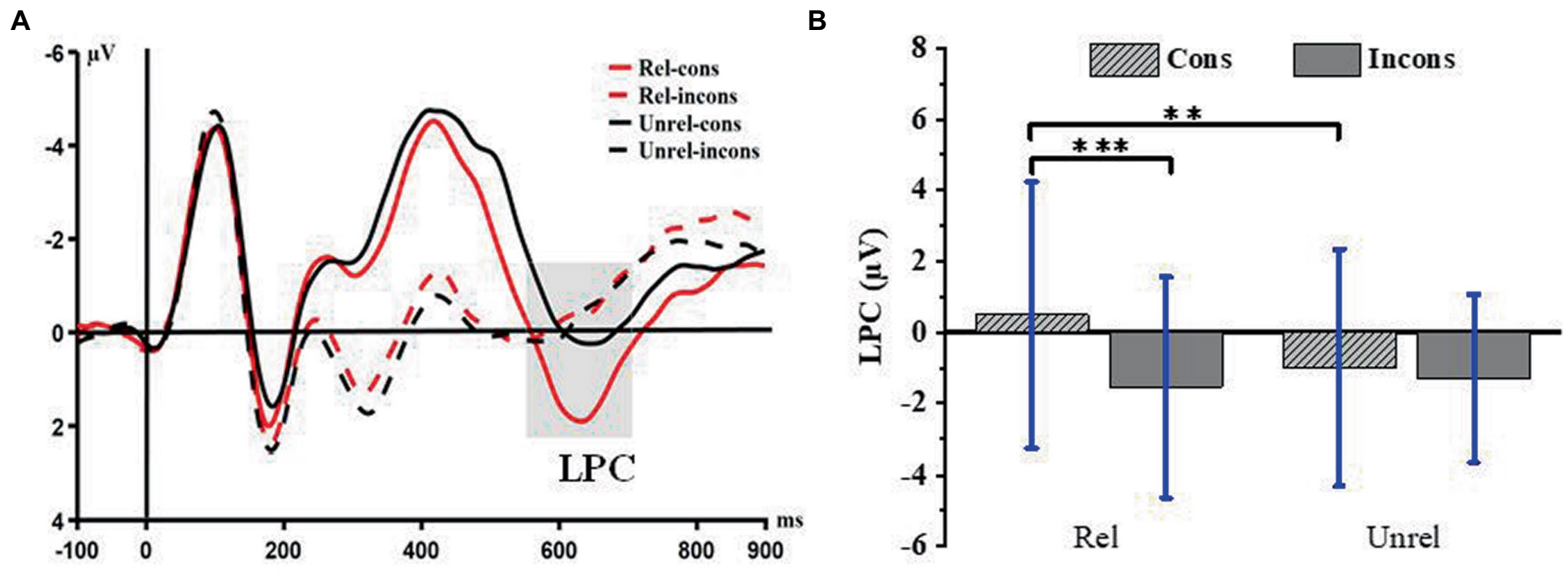
**(A)** Late positive components (LPC) time-locked to the four conditions. Event-related potentials (ERPs) were calculated by averaging the data at the electrodes of Fz, FC5, FC6, CZ, C3, C4, and PZ. **(B)** Histogram in conditions, bars represent standard deviation of the mean, **p < 0.01, ***p < 0.001. Rel, related; Unrel, unrelated; Cons, consistent; Incons, inconsistent.

The average amplitude of LPC in the 550–700 ms time window was analyzed by repeated measurement variance of 2 (relatedness: related lures, unrelated lures) × 2 (consistency: consistent lures, inconsistent lures). The results showed that the main effect of relatedness is not significant [*F*(1,27) = 3.18, *p* = 0.09], and there was no significant difference between the average amplitudes of LPC evoked by the related lures (−0.53 μ V ± 3.30) and the unrelated ones (−1.15 μV ± 2.67). The main effect of consistency is significant [*F*(1,27) = 12.84, *p* = 0.001, η_p_^2^ = 0.32], and the average amplitude of LPC evoked by consistent lures (−0.25 μV ± 3.36) was significantly higher than that induced by inconsistent (−1.42 μV ± 2.54). The interaction between relatedness and consistency is significant [*F*(1,27) = 13.77, *p* = 0.001, η_p_^2^ = 0.34]. Further simple effect analysis showed that under related conditions, the average amplitude of LPC induced by consistent lures (0.49 μV ± 3.75) is significantly larger than that induced by inconsistent (−1.54 μV ± 3.10); *F*(1,27) = 28.90; *p* < 0.001; η_p_^2^ = 0.52. This shows that there is neighborhood consistency in simple multiplication arithmetic, and related consistent lures are expected when the credibility of the correct answer is lower. Regarding the condition of unrelated, there is no significant difference between consistent and inconsistent lures [*F*(1,27) = 0.56, *p* = 0.46]. Under the consistent condition, the amplitude of LPC evoked by related lures (0.49 μV ± 3.75) was significantly larger than that induced by unrelated (− 0.99 μV ± 3.33); *F*(1,27) = 11.92; *p* = 0.002; η_p_^2^ = 0.31. This shows that the activation diffusion of arithmetic problems will activate not only the correct answers but also the related wrong lures, and extracting related wrong lures may require more cognitive resources. Regarding the condition of inconsistency, there was no significant difference between related and unrelated lures [*F*(1, 27) = 0.36, *p* = 0.57]. Detailed waveforms and bar charts are shown in Figure 3.

## 4. Discussion

This study aimed to investigate whether relatedness and consistency affect individuals’ solutions to multiplication problems for multiplicative probes in the auditory channel. Using the delayed verification paradigm, participants were required to judge whether the probes were correct or not. The behavioral and ERP results showed that related consistent lures had faster responses than the related inconsistent ones and induced larger N400 and LPC average amplitudes. This shows neighborhood consistency in the mental calculation of simple multiplication under the presentation of the auditory channel, and the related consistent lures can promote the mental multiplication calculations.

### 4.1. Significance of component identification

N400 is a negative component observed at the end when the participants read words and sentences with inconsistent semantics (Kutas and Hillyard, 1989). The amplitude of the N400 effect is inversely proportional to the amount of activation diffusion from the arithmetic problem (Friederici, 1995; Niedeggen and Rösler, 1999). This suggests that if the target word is preceded by a relevant context (word or sentence), the amplitude is smaller, while if there is an irrelevant context before the target word, the amplitude is larger. Compared with unrelated inconsistent lures, the activation of related consistent lures is less likely to extend to other results. This difference in activation diffusion may be due to the active inhibition of digitally unreliable representations (Campbell and Graham, 1985). The study found that related consistent lures induced larger N400 components than related inconsistent. This shows that related consistent lures are less affected by the diffusion of activation of arithmetic problems and can be judged more quickly.

Late positive components is related to stimulus recognition, semantic processing, and credibility judgment (Polich, 2012). First, among the lures, consistent lures induce larger LPC amplitudes than inconsistent ones, indicating that the processing of decade consistent lures is more likely to create wrong judgments, determined by the representation of two-digit decomposition processing. In the numerical size judgment task, even if the participants can react by comparing one of the two digits, they will still compare decade and unit digits simultaneously, aligning with several studies (Nuerk et al., 2001; Kang et al., 2007; Moeller et al., 2013; Faulkenberry et al., 2020). Second, the amplitude of LPC evoked by the related consistent lures is significantly larger than that of the related inconsistent ones, indicating that the related consistent ones are perceived as the correct answer with lower credibility and are easier to judge.

Our results show that when the auditory channel presents arithmetic, there is no significant difference between the related lures and the unrelated ones in response time, accuracy, N400 and LPC. It shows that the speech strategy used by individual memory arithmetic (Zhou et al., 2007; Cheng et al., 2017) strengthens the relationship between phonetic numbers and arithmetic memory, so that arithmetic information is processed differently at different levels of representation, which is consistent with recent studies. That is, the similarity between coding and retrieval (the initial information acquisition channel is the same as the test channel) promotes memory retrieval (Dickson et al., 2018; Zhu et al., 2019). In addition, the results show that the reaction time of the related consistent lures is significantly faster than that of the related unconsistent ones, and the N400 and LPC amplitudes induced by the related consistent lures are larger, indicating that the adjacent consistent answer can promote simple multiplication. This is inconsistent with the results of Domahs et al. (2007), who found that the average amplitudes of N400 and LPC induced by related unconsistent ones are larger. This may be affected by the presentation time of different operands. Some studies have found that the relevant lures can be activated only with sufficient presentation time (Lemaire and Fayol, 1995). In the study of Domahs et al. (2007), all operands present 100 ms, which may lead to inconsistent lures are less related to the presentation question and less reliable. This leads to larger N400 and LPC amplitudes.

### 4.2. Study’s significance

This study supports the INM. The model believes that arithmetic probes retrieval includes two-layer processing, promoting cooperation and suppressing competition. If the presented probes are related to operands and share the same decade digits as the correct result, they will improve the fluency of arithmetic retrieval and thus promote the response to the probes. In contrast, if the probes are related to the operand but the decade digits differ, the response is suppressed. The results are identical to the model prediction; that is, the average amplitude of N400 and LPC of the related consistent lures is larger than that of the related inconsistent ones. Additionally, the results show that, compared with Domahs et al. (2007), there is no significant difference in relatedness under the probes in auditory channel presentation, which may be because the difference in activation and diffusion of multiplication arithmetic only depends on the presentation of visual channel. In other words, for the auditory representation of arithmetic probes, there may be a stronger semantic relationship between the multiplication facts, but the activation difference between related and unrelated lures is not as significant as predicted by Verguts and Fias (2005a).

Number sense—the ability to perceive numbers—is an important part of mental arithmetic. It includes sensitivity to the relationship between numbers and proficiency in identifying unreliable digital probes (Berch, 2005). The development of number sense helps individuals cultivate the ability to apply what they have learned to new problems (Hatano and Oura, 2003). This study found that the N400 and LPC amplitudes of the related consistent lures were significantly larger than those of the related inconsistent ones, indicating that the participants were very sensitive to the characteristics of arithmetic numbers, which further showed that the related consistent lures were recognized as correct answers with lower credibility. Therefore, as a part of number sense, the electrophysiological study of neighborhood consistency of simple multiplication mental calculation strengthens our grasp of the law of number sense further.

### 4.3. Limitations and future suggestions

This study has a few limitations. First, regarding the presentation frequency of multiplication formulas, each multiplication problem was presented 24 times in the experiment, wherein the correct answer was presented 12 times, and the four conditions of the wrong lures were presented 3 times each. This arrangement was to ensure that the average number of superimposed segments of all participants under each error condition was more than 30. However, frequently presented problems may be solved more accurately (Bourne and Birbaumer, 1998), leading to a specific practice effect and affecting the speed at which individuals integrate the problems into the retrieval network. Second, although the study provides electrophysiological evidence of related consistent lures and related inconsistent ones from the perspective of arithmetic lures in the auditory channel, channel specificity is not used as a direct operating variable to investigate its effect on neighborhood consistency. Hence, the causal relationship between channel form and simple multiplicative mental arithmetic judgment cannot be determined.

Rotem and Henik (2015) examined the sensitivity of children with and without MLD to multiplicative arithmetic-related and consistent digital features. They found that children are first sensitive to Operand-related numerical features (children without MLD in Grade 3 and children with MLD in Grade 8), and then sensitive to decade digits consistent numerical features (children without MLD start in Grade 4 and tend to mature the pattern in Grade 6), indicating a difference of sensitivity to numerical features in different ages. Therefore, future research can investigate the age difference of multiplication mental arithmetic in network memory in the process of individual development and further clarify the cognitive neural mechanism of neighborhood consistency. Additionally, although the interference effect in the extraction process has been supported, the interference effect in the problem presentation process requires further attention. In other words, most studies have focused on the ERPs triggered by the presentation of the probes to arithmetic facts, and few have examined the cognitive neural characteristics of the processing process of the arithmetic problem (Szűcs and Csépe, 2004; Muluh et al., 2011; Jasinski and Coch, 2012). According to the INM, the representation of an arithmetic problem will activate not only the correct results but also the related wrong lures; hence, the cognitive neural mechanism of the brain must be further examined when different arithmetic problems are presented.

## 5. Conclusion

This study found that the reaction time of the related consistent lures is significantly faster than that of the related inconsistent ones, and the amplitudes of N400 and LPC induced by the related consistent lures are significantly larger than those of the related inconsistent ones. The wrong lures relating to operands and sharing the same decade digits with the correct answer therefore accelerate the judgment of multiplication mental arithmetic.

## Data availability statement

The original contributions presented in the study are included in the article/supplementary material, further inquiries can be directed to the corresponding author.

## Ethics statement

The studies involving human participants were reviewed and approved by the Ethics Committee of the College of Psychology of Guizhou Normal University. The patients/participants provided their written informed consent to participate in this study.

## Author contributions

YP, JS, LC, LJ, and WT were in charge of literature review, data collection, data analysis, and writing. YP and HY ensured supervision over selecting the topic and research design. All authors contributed to the article and approved the submitted version.

## Funding

This study was supported by the National Natural Science Foundation of China (no. 0518075).

## Conflict of interest

The authors declare that the research was conducted in the absence of any commercial or financial relationships that could be construed as a potential conflict of interest.

## Publisher’s note

All claims expressed in this article are solely those of the authors and do not necessarily represent those of their affiliated organizations, or those of the publisher, the editors and the reviewers. Any product that may be evaluated in this article, or claim that may be made by its manufacturer, is not guaranteed or endorsed by the publisher.
